# A pan-cancer analysis of MARCH8: molecular characteristics, clinical relevance, and immuno-oncology features

**DOI:** 10.1080/15384047.2025.2458773

**Published:** 2025-01-29

**Authors:** Zihan Quan, Songqing Fan, Hongmei Zheng, Yue Ning, Yang Yang

**Affiliations:** a Department of Pathology, The Second Xiangya Hospital, Central South University, Changsha, Hunan, China; b Department of Pathology, The Fourth People’s Hospital of Longgang District, Shenzhen, Guangdong, China

**Keywords:** MARCH8, pan-cancer analysis, tumor immune infiltrating, E3-ubiquitin ligase, NSCLC

## Abstract

Membrane-associated RING-CH8 (MARCH8) is a member of the recently discovered MARCH family of ubiquitin ligases. MARCH8 has been shown to participate in immune responses. However, the role of MARCH8 in prognosis and immunology in human cancers remains largely unknown. The expression of MARCH8 protein was detected via immunohistochemistry in non-small cell lung cancer (NSCLC) and non-cancerous lung tissues. The study investigated the role of MARCH8 in tumor immunity through pan-cancer analysis of multiple databases. MARCH8 genetic alternations and expression were explored with the cBioPortal, GTEx, and TCGA databases. We investigated the role of MARCH8 expression in clinical relevance, prognosis, tumor immune microenvironment, immune checkpoint (ICP) with a series of bioinformatics tools and methods, such as TISIDB database, ESTIMATE, and CIBERSORT method. MARCH8 expression showed cancer-specific dysregulation and was associated with the prognosis of patients in various cancers. MARCH8 was related to the tumor microenvironment and participated in tumor immune regulation. Furthermore, low expression of MARCH8 was associated with poor prognosis and might serve as an independent prognostic biomarker for NSCLC patients. The comprehensive pan-cancer analysis revealed the potential of MARCH8 in tumor-targeted therapy, and suggested MARCH8 as a promising prognostic and immunological pan-cancer biomarker.

## Introduction

Post-translational modification (PTM) of proteins refers to the chemical modifications that occur after protein biosynthesis and are related to various protein functions, such as activity and expression level, subcellular localization, and protein interactions.^1^ PTMs are complex processes, including phosphorylation, glycosylation, ubiquitination, acetylation, and methylation.^2^ Protein ubiquitination is one of the most common PTMs that plays important roles in organelle biosynthesis, DNA repair, protein transport, and immune response.^3,4^ Protein ubiquitination consists of a series of complicated processes that are collectively catalyzed by E1 (ubiquitin-activating enzyme), E2 (conjugating enzyme), and E3 (ubiquitin ligase). E1 activates the ubiquitin and then transfers it to E2 in an ATP-dependent manner, after which E3 binds to the ubiquitin and transfers it to a primary amine of the target proteins. E3 ligases have been extensively researched for the role in dictating substrates specificity.^5,6^ They are categorized into three major types based on their structural properties: including the really interesting new gene (RING), the homologous to the E6-AP carboxyl terminus (HECT), and the RING-between-RING (RBR) families.^7^

Membrane-associated RING-CH (MARCH) family proteins are a subfamily of the RING-finger E3 ligases, characterized by a C4HC3 cysteine-histidine (RING-CH finger) domain.^8^ Recent studies have reported that MARCH proteins regulate immune responses by catalyzing polyubiquitination of various immune receptors.^8,9^ MARCH8 is the first identified member of the MARCH family that plays a significant role in the immune response.^10^ Aberrant expression of MARCH8 has been shown to downregulate a variety of immunomodulatory proteins, such as major histocompatibility complex II (MHC II), CD166, CD88, and CD98.^11,12^ MARCH8 is also a newly identified tumor suppressor that inhibits breast cancer metastasis and enhances cancer cell death.^13^ Furthermore, MARCH8 has been reported to be associated with the growth of the esophageal tumors and non-small cell lung cancer (NSCLC).^14,15^ However, the role of MARCH8 in tumor prognosis and tumor immunology remains obscure.

This study investigated the role of MARCH8 in the progression of NSCLC. We systematically analyzed the mechanism of influence and prognostic and immunotherapy value of MARCH8 expression across cancer types. With multiple databases, our pan-cancer analysis explored the association of MARCH8 genetic alteration, expression, clinical relevance, prognosis, tumor microenvironment (TME), and immune checkpoint (ICP) genes across cancer types. Our data highlight the potential significance of MARCH8 in tumor development, prognosis, and the TME, and suggest strategies that could promote collaborative effects in the context of immunotherapy.

## Materials and Methods

### Genetic alterations and expression analysis of MARCH8 in pan-cancer

We downloaded the uniformly standardized pan-cancer dataset (TCGA TARGET GTEx PANCAN), and extracted the expression data of the MARCH8 gene in each tumor and normal tissue from the UCSC database (http://xenabrowser.net/). We used the cBioPortal database (https://www.cbioportal.org) to analyze the uniformly standardized TCGA Pan-Cancer dataset for mutations, copy number alterations (CNAs), and gene fusions data.^16^ We also downloaded and integrated the gene-level Copy Number Variation (CNV) dataset and gene expression data of Level 4 for all TCGA samples treated by GISTIC software (DOI: 10.1186gb-2011-12-4-r41/) from GDC (https://portal.GDC.cancer.gov/).

We calculated the differences in gene expression between tumor and normal tissues in each sample by R software. The correlations between MARCH8 expression and immune, molecular subtypes, clinical stage, and tumor grade in different cancer types were explored via the TISIDB database (http://cis.hku.hk/TISIDB/index.php).^17^ All of the gene expression matrix were performed the log2(x + 0.001) transformation and removed for batch effects with ComBat-seq.^18^ *P*-value < .05 was considered statistically significant.

### Prognostic analysis of MARCH8 in pan-cancer

We conducted a univariate survival analysis to examine the correlation between the expression level of MARCH8 with patient prognosis, which included overall survival (OS), disease-free interval (DFI), disease-free survival (DSS), and progression-free interval (PFI). The Kaplan-Meier method was used to compare survival with the high and low expression levels of MARCH8. Cancer types with the *P*-value > .05 were excluded from the analysis.

### Association analysis of MARCH8 with tumor immune cell/infiltration and ICP genes

To investigate the role of MARCH8 in tumor infiltration, we analyzed the correlation between MARCH8 and tumor infiltration cells in each cancer type. The tumor infiltration scores of 22 immune cells in each tumor based on gene expression were evaluated based on the CIBERSORT method.^19^ And the stromal, immune, and ESTIMATE scores of each tumor were calculated by R package ESTIMATE.^20^ The marker genes of the immune cells with Benjamini-Hochberg adjusted *P*-value < .01 were considered statistically significant. Further, we explored the relationship between MARCH8 expression and ICP genes, considering correlations significant when the *P*-value < .05.

### Tissues

This study was approved by The Second Xiangya Hospital of Central South University Ethics Review Board (Scientific and Research Ethics Committee, No. K021/2021). In this study, we collected 339 cases of NSCLC tissue and 80 cases of non-cancerous lung tissue from The Second Xiangya Hospital of Central South University (Changsha, China). Complete follow-up data and clinical records were available for all patients. Each case had a definite clinical stage and pathological diagnosis based on the Eighth Edition Lung Cancer Stage Classification and the WHO histological classification.^21^ All patients involved in our study written informed consent. We used the tissue microarrays (TMA) technology to design and construct high-throughput NSCLC TMAs according to the protocol previously described.^22^

### Immunohistochemistry (IHC) and scores

The IHC experiment and evaluation method were conducted following the protocol of our former study.^23^ The dilution of primary antibody MARCH8 (14119–1-AP, Proteintech) was 1:500. The IHC score was independently performed under 200× light microscopy by YY and SF who were blinded to the clinicopathological data. The total IHC score = intensity score×percentage score. The staining intensity of MARCH8 was scored as 3 (strong), 2 (moderate), 1 (weak), and 0 (negative), besides, staining percentage was scored as 4 (76–100%), 3 (51–75%), 2 (26–50%), 1 (1–25%), and 0 (0%). The scores of MARCH8 proteins ranged from 0 to 12, and the optimal cutoff levels were 4, based on the OS of NSCLC patients using the log-rank test. MARCH8 was divided into low/high expression. All differences in scores were resolved through discussion, achieving 95% consistency between the two evaluators.

### Statistical analysis

Data were analyzed using R (version 3.6.4), GraphPad Prism (version 9.0), and SPSS (version 26.0). Differences among groups were analyzed by Wilcoxon Rank Sum and Signed Rank Tests between two individual groups, and by Kruskal-Wallis tests for multiple groups. The Kaplan-Meier curves were used to assess survival outcomes, and correlations were evaluated with Spearman’s correlation coefficients. Univariate analysis and multivariate models were performed with the Cox proportional-hazards regression model by SPSS. A two-sided *p* < .05 was the threshold of significance.

## Results

### Genetic alterations and expression pattern of MARCH8 across cancer types

**Figure 1.**
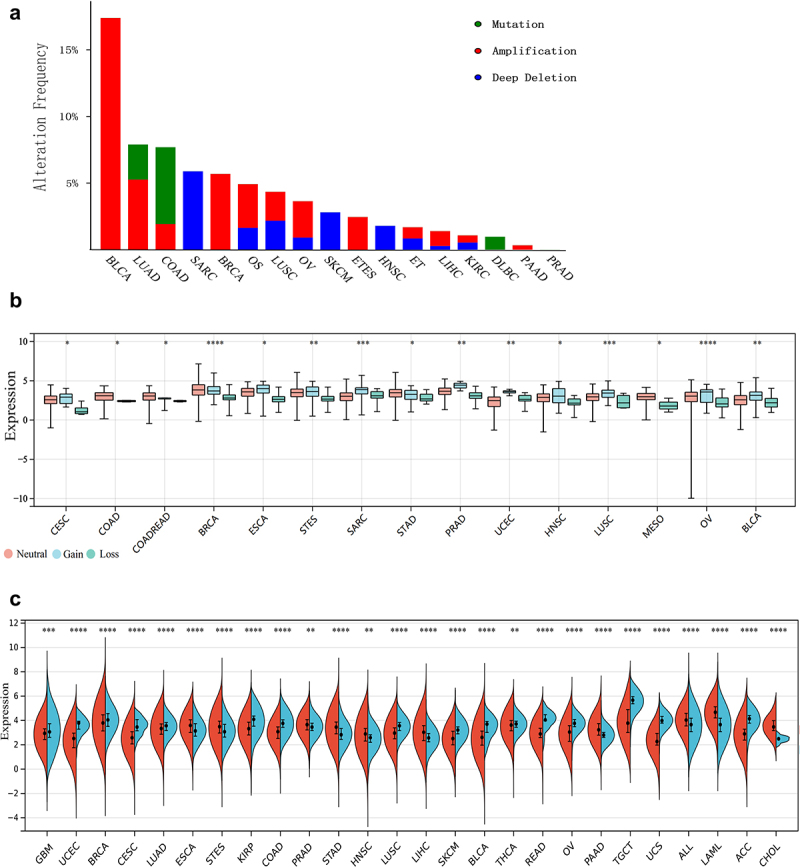
Genetic alterations and expression pattern of MARCH8 across cancer types. (a) Distribution of the CNA alterations of MARCH8 in cancers harboring the highest alteration rates (green: mutation, red: amplification, blue: deep deletion). (b) The CNV alteration expression (Mean±sd) distribution of MARCH8 across cancer types. (c) The mRNA expression of MARCH8 gene across cancer types (red) and normal tissues (blue). Differences among groups were analyzed by Wilcoxon rank sum and signed rank tests between two individual groups, and by Kruskal-Wallis tests for multiple groups. ^−^*p* > .05, *p < .05, **p < .01, ***p < .001, ****p < .0001.

CNVs are the hallmarks in the cancer genome and are important somatic genomic variants that contribute to tumorigenesis. We observed the genetic alterations status of MARCH8 across cancer types and found that gene amplification was the most frequent type across the various cancers (Figure 1a). Applying CNV analysis, we observed widespread CNV alteration of MARCH8 across cancers, among which amplification and deletion vary (Figure 1b). MARCH8 displayed the prevalent CNV amplification in most cancers like ovarian serous cystadenocarcinoma (OV) and bladder urothelial carcinoma (BLCA) (The cancer names and their abbreviations were referenced from TCGA database listed in Table S1).

We further explored the mRNA expression of MARCH8 in various cancers. As shown in Figure 1c, the mRNA expression level of MARCH8 exhibited tumor heterogeneity. Compared to the normal tissues, MARCH8 mRNA expression level was significantly higher in digestive system tumors. However, MARCH8 expression was significantly lower in reproductive and urological tumors like testicular germ cell tumor (TGCT) and uterine corpus endometrial carcinoma (UCEC). Interestingly, MARCH8 mRNA expression was higher in lung adenocarcinoma (LUAD) but lower in lung squamous cell carcinoma (LUSC) compared to normal lung tissues. However, MARCH8 displayed CNV amplification in both LUAD and LUSC.

### The relationship between MARCH8 expression and immune, molecular subtypes, and clinical features across cancer types

**Figure 2.**
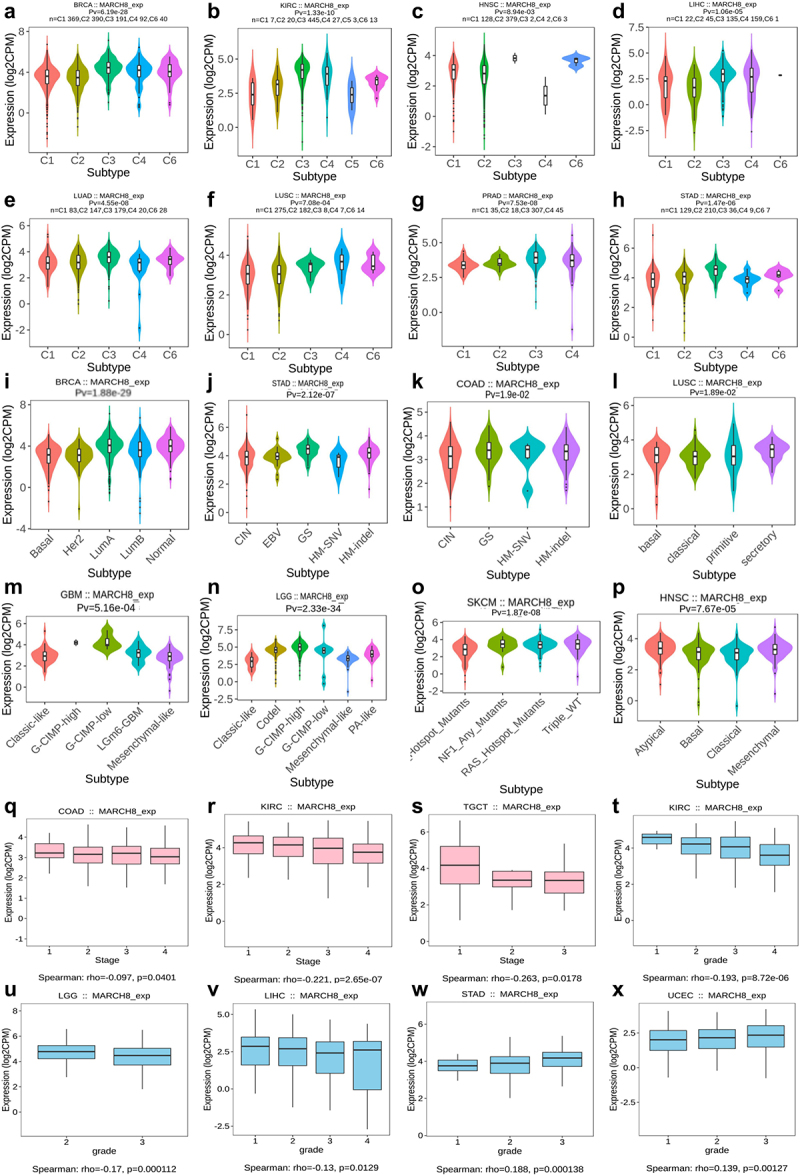
The relationship between MARCH8 expression and different immune subtypes and molecular subtypes across cancer types. (a-p) The relationship between MARCH8 expression and different immune subtypes across cancer types. (q-x) The relationship between MARCH8 expression and different molecular subtypes across cancer types. (Spearman’s correlation, all *p* < .05).

Next, we explored the role of MARCH8 expression on immune and molecular subtypes among human cancers in the TISIDB dataset. Immune subtypes were classified into six types, including C1 (wound healing), C2 (IFN-gamma dominant), C3 (inflammatory), C4 (lymphocyte depleted), C5 (immunologically quiet), and C6 (TGF-gamma dominant).^24,25^ Figure 2a illustrated that MARCH8 expression was related to different immune subtypes in most cancers like breast invasive carcinoma (BRCA) and brain lower grade glioma (LGG). MARCH8 showed high expression in C3, C4, and C6 immune types compared to C1 and C2 types in BRCA (Figure 2b). For different molecular subtypes of cancers, the significant connection with MARCH8 expression also existed in LGG and BRCA (Figure 2c,d). We also explored the associations between MARCH8 and clinical features (Figure S1). The results showed MARCH8 gene was associated with lower tumor stages in kidney renal clear cell carcinoma (KIRC). And MARCH8 expression was associated with lower grades in KIRC but higher tumor grades in stomach adenocarcinoma (STAD). Based on the above results, we concluded that MARCH8 expression differs in immune, molecular subtypes, clinical stages, and tumor grades across cancer types.

### The prognostic potential of MARCH8 across cancer types

**Figure 3.**
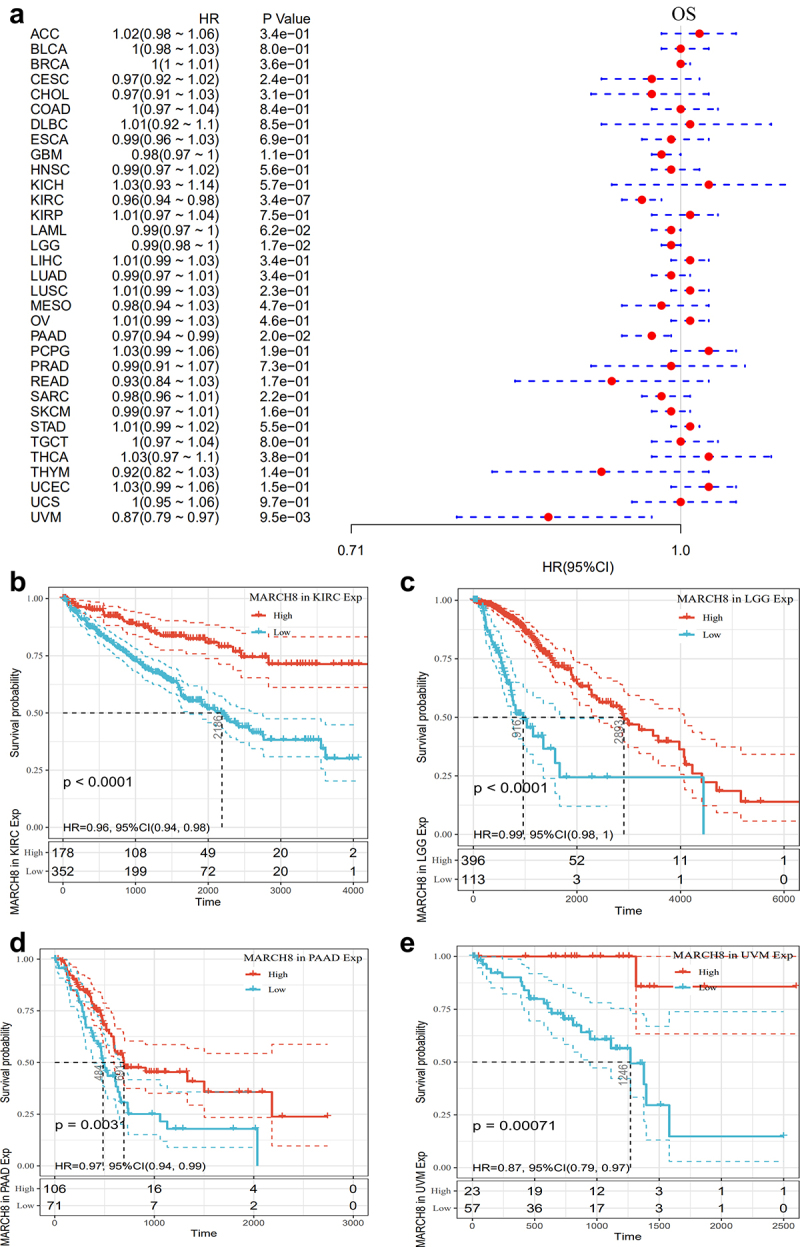
The prognostic potential of MARCH8 across cancer types. (a) The relationship between MARCH8 expression and OS across cancer types, using univariate survival analysis (only *P*-values < 0.05 shown). Kaplan-Meier OS curves of MARCH8 expression in KIRC (b) and LGG patients (c).

To investigate the prognostic potential of MARCH8 in human cancers, we performed univariate Cox regression analyses based on expression data (median as the cutoff to divide the patients into the higher and lower group) data from pan-cancer of TCGA. As shown in Figure 3a, the forest plots and survival map indicated that MARCH8 expression correlated with the OS of various tumor patients. The KM curves for tumors in which MARCH8 expression was significantly associated with the patient outcome were shown in Figure 3b,c. The results showed that higher expression of MARCH8 was associated with poorer outcome for KIRC and LGG patients. The relationship between MARCH8 and DFI and DSS analyzed by univariate Cox regression were shown in Figure S2. These observations revealed that MARCH8 might function in different roles in the outcomes across cancer types. Moreover, higher expression of MARCH8 predicted poorer prognosis in patients with LUSC, while increased MARCH8 expression was associated with prolonged OS in LUAD.

### The correlation of MARCH8 expression with tumor immune cell infiltration, immune checkpoint genes across cancer types

Based on MARCH8 expression differing in tumor immune subtypes, we explored the role of MARCH8 expression in TME across cancer types. Initially, we investigated the correlation between MARCH8 expression and the tumor immune infiltrating score of individual tumor samples using the R package Estimate. The results indicated that MARCH8 expression was correlated with the level of tumor cell immune infiltration across cancer types. MARCH8 expression was significantly positively correlated with the Immune Score and Stromal Score in urinary and digestive system cancers, but negatively in reproductive system tumors.

**Figure 4.**
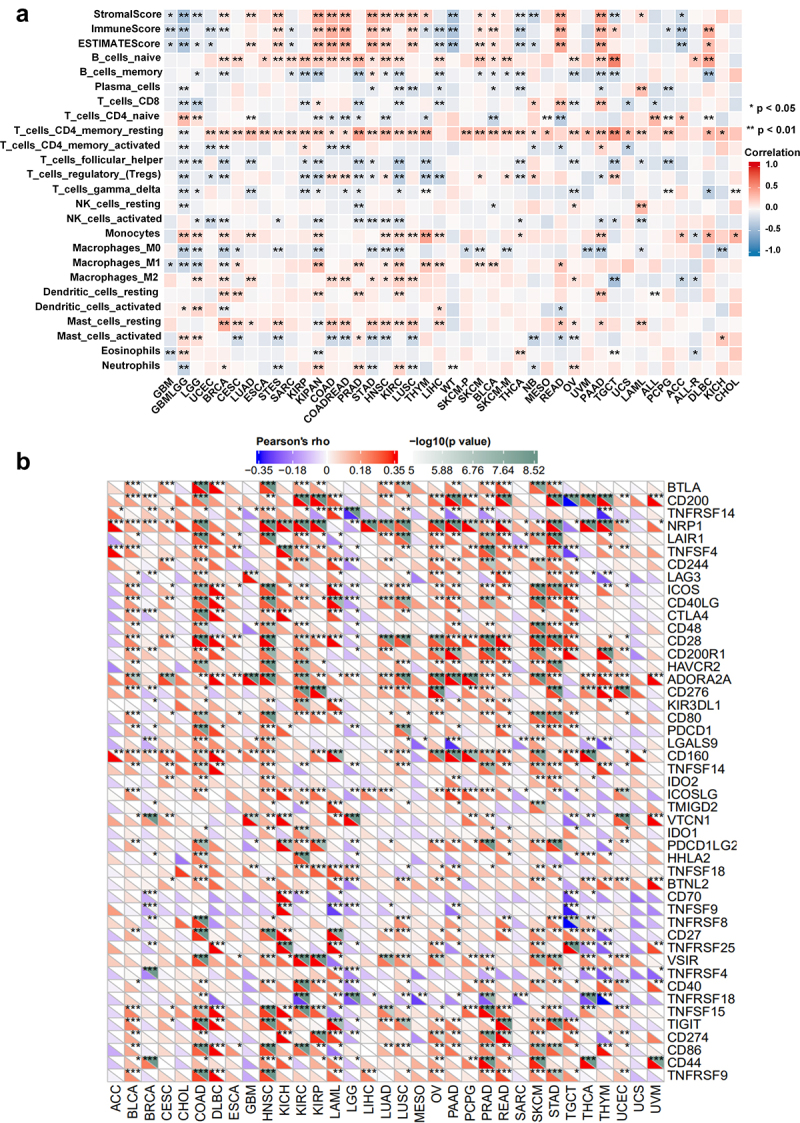
Correlation analysis between expression of MARCH8 and immune cell infiltration and ICP genes across cancer types. (a) Correlation analysis between expression of MARCH8 and immune cells across cancer types by CIBERSORT, and immune infiltration score based on estimate. (b) Correlation analysis between expression of MARCH8 and ICP gene expression across cancer types. *p < .05, **p < .01, ***p < .001.

Next, we analyzed the connection between MARCH8 expression and immune cell infiltration levels of 22 immune cells by CIBERSORT (Figure 4a, BH-adjusted *p* < .01). Notably, MARCH8 expression was highly positively correlated with CD4+ T memory resting cells in 33 cancer types, but negatively in glioma (GBMLGG). MARCH8 expression was also highly positively correlated with B naive cells but negatively correlated with B memory cells in most cancer types. Furthermore, MARCH8 expression was negatively correlated with M0 Macrophages but positively correlated with M1 Macrophages in most cancers.

Studies have reported that ICP genes influence on immune cell infiltration and immunotherapy.^26^ Subsequently, we explored the associations between MARCH8 expression and ICP genes across cancer types (Figure 4b). Our results indicated strong relationships between MARCH8 expression and ICP genes in several cancer types, such as colon adenocarcinoma (COAD), head and neck squamous cell carcinoma (HNSC), and skin cutaneous melanoma (SKCM). Together, these results demonstrated that MARCH8 expression is correlated with immune infiltration of human cancers.

### The role of MARCH8 protein in NSCLC tumor progression

**Figure 5.**
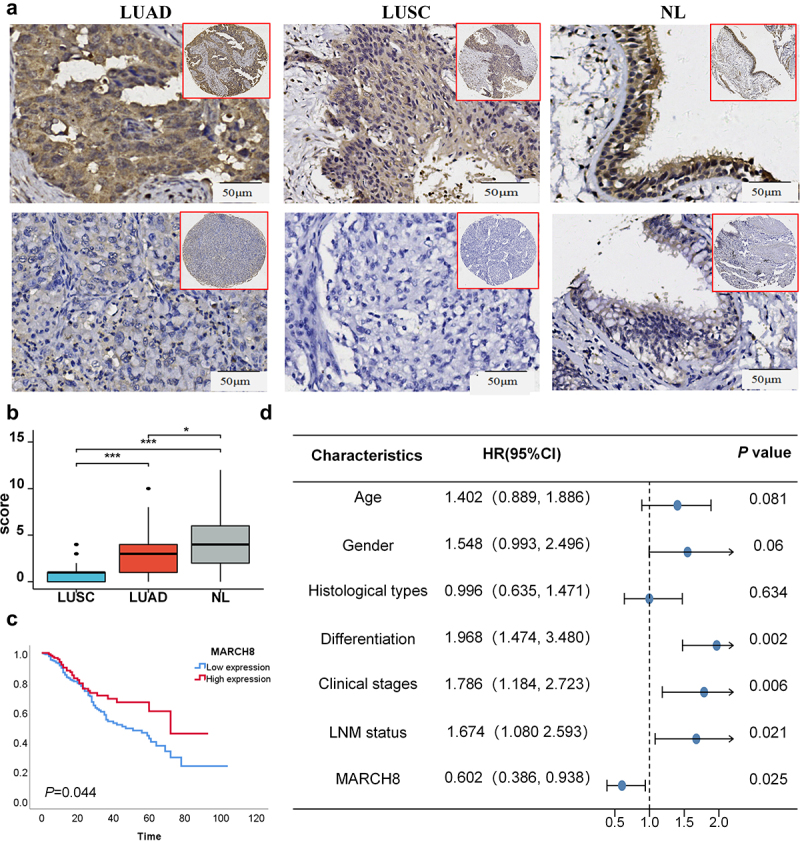
The expression of MARCH8 protein and prognosis potential in NSCLC tissues. (a) The expression of MARCH8 protein in LUAD, LUSC, and noncancerous lung tissues detected by IHC (DAB staining, original magnification 200×and 40×). (b) The expression of MARCH8 in LUSC, and LUAD compared to the noncancerous lung tissues. *p < .05, ***p < .001. (c) Kaplan-Meier overall survival analysis of NSCLC patients based on MARCH8 proteins expression (log-rank test, *p* = .044). (d) The univariate survival analysis of OS and MARCH8 protein expression and clinical-pathological features in NSCLC patients.

Based on the tumor heterogeneous expression of MARCH8 mRNA in LUAD and LUSC, we further detected the expression and subcellular localization of MARCH8 protein in NSCLC tissues and non-cancerous lung tissues by IHC. The positive expression of MARCH8 protein was predominantly discovered in the cytoplasm of cells and rarely identified in the nucleus (Figure 5a). Contrary to the mRNA expression levels, MARCH8 protein expression levels were reduced in LUSC and LUAD compared to normal lung tissues (Figure 5b).

We further assessed the association between clinicopathological features and MARCH8 protein expression in NSCLC by chi-square test. As shown in Table 1, low expression level of MARCH8 protein was positively correlated with the differentiation, histological type, and lymph node metastasis (LNM) of NSCLC patients (*p =* 0.008, *p* < .001, *p* = .002, respectively). However, no significant relationship was found with age, gender, and clinical stage of NSCLC patients (all *p* > .05).

**Table 1.** Correlation between MARCH8 protein expression and the clinicopathologic characteristics of NSCLC patients (*n* = 339).

Clinicopathologic characteristics(n)	MARCH8		
	L(%)	H(%)	*p-*Valve
Age (years)			
<60 (n = 222)	149(67.1)	73(32.9)	0.073
≥60 (n = 117)	67(27.4)	50(42.7)	
Gender			
Male (n = 253)	156(61.7)	97(38.3)	0.177
Female (n = 86)	60(69.8)	26(30.2)	
Differentiation			
Well/Moderate (n = 139)	77(55.4)	62(44.6)	0.008*
Poor (n = 200)	139(69.5)	61(30.5)	
Histological types			
LUSC (n = 165)	87(52.7)	78(47.3)	0.000*
LUAD (n = 174)	129(74.1)	45(25.9)	
LNM status			
No LNM (n = 150)	109(72.7)	41(27.3)	0.002*
LNM (n = 189)	107(56.6)	82(43.4)	
Clinical stages			
Stage I,II (n = 177)	118(66.7)	59(33.3)	0.268
Stage III (n = 161)	98(60.9)	63(39.1)	

LUAD, lung adenocarcinoma; LUSC, lung squamous cell carcinoma; LNM, lymph node metastasis. *p < .05.

Subsequently, we used Kaplan‐Meier survival analysis to explore the prognostic value of increased expression of MARCH8 protein in NSCLC. As Figure 5c showed that lower expression level of MARCH8 protein was related to the poorer OS of NSCLC patients. Moreover, we investigated both univariate and multivariate Cox regression analyses to validate whether MARCH8 protein could serve as the independent prognostic biomarkers in NSCLC patients (Figure 5d). The results in Table 2 presented that low expression of MARCH8 protein could serve as an independent prognostic biomarker for NSCLC patients (*p=*0.021).

**Table 2.** Correlation between MARCH8 protein expression and characteristics in NSCLC patients via univariate and multivariate Cox analysis.

Characteristics	Univariate			Multivariate		
	Survival time(SE)	95%CI	*P-*Valve	Exp(B)	95%CI	*P-*Valve
Age						
<60	59.76 (4.03)	(51.86, 67.65)	0.056	1.402	(0.889, 1.886)	0.081
≥60	49.92 (4.28)	(41.68, 58.18)				
Gender						
Male	54.99 (3.83)	(47.48, 62.50)	0.090	1.548	(0.993, 2.496)	0.060
Female	60.83 (4.48)	(52.05, 69.62)				
Histological types						
LUAD	52.13 (3.27)	(45.73, 58.53)	0.696	0.996	(0.635, 1.471)	0.634
LUSC	63.42 (4.71)	(54.19, 72.65)				
Differentiation						
Well/Moderate	68.81 (4.04)	(55.89, 71.72)	0.000*	1.968	(1.474, 3.480)	0.002*
Poor	51.26 (3.75)	(43.91, 58.60)				
Clinical stages						
Stage I,II	70.93 (4.49)	(62.13, 79.73)	0.000*	1.786	(1.184, 2.723)	0.006*
Stage III	42.69 (2.96)	(36.89, 48.49)				
LNM status						
LNM	44.11 (4.54)	(38.98, 49.24)	0.000*	1.674	(1.080 2.593)	0.021*
No LNM	69.67 (2.61)	(60.76, 78.57)				
MARCH8						
Low expression	53.31 (3.62)	(46.21, 60.40)	0.019*	0.602	(0.386, 0.938)	0.025*
High expression	63.49 (4.68)	(54.32, 72.66)				

CI, confidence interval; SE, standard error; Exp(β), odds ratio; LUAD, lung adenocarcinoma; LUSC, lung squamous cell carcinoma; LNM, lymph node metastasis. *p < .05.

## Discussion

Growing evidence indicates that ubiquitination, an important process for post-translational regulation of proteins, plays different roles in cancer diseases. MARCH8, a member of the MARCH family proteins, is part of the RING E3 groups but possesses a unique RING-CH domain, distinguishing it from the classic RING domain.^8,27^ MARCH8 has been found to be dysregulatly expressed and exert anti-tumor activities in many cancer types, including NSCLC and esophageal cancer.^13–15^ MARCH8 also reduces the surface expression of ligand receptor 1 (TRAIL-R1) in breast cancer cells,^28^ indicating the presence of different substrates in a cell context-dependent way. The abnormal expression of MARCH8 leads to the downregulation of several immunomodulatory receptors, such as MHC I, HLA 2.1, and TNF-related apoptosis,^28,29^ indicating its potential as a promising target in cancer immunotherapy. Initial studies of the MARCH8 have primarily focused on its immunomodulatory role, but its relevance in each cancer type has not been fully elucidated. Therefore, our study is the first to provide a comprehensive pan-cancer analysis of MARCH8, examining its genetics, expression, prognosis, and immunity across cancer types. We further explored the specific role of MARCH8 in NSCLC using IHC.

In the first step of our study, we analyzed the genomic alternation, CNVs, and expression of MARCH8 across cancer types. The epigenomic and genomic changes may be responsible for the cancer-specific dysregulation of MARCH8 in cancer. Moreover, MARCH8 mRNA expression levels were significantly higher in digestive system tumor, while significantly lower in reproductive and urological tumors. Furthermore, MARCH8 expression differed within immune and molecular subtypes, clinical stages, and grades in most cancer types such as LGG and BRCA. Consistent with previous studies, patients with lower MARCH8 expression patients had a worse prognosis in BRCA.^13,15^ In addition, in many other cancer types such as KIRC and LGG, higher MARCH8 expression meant the poorer prognosis. Take together, MARCH8 expression might regulate cancer progression.

Overexpression of MARCH8 has been found to inhibit NSCLC cell proliferation and metastasis via the phosphoinositide 3-kinase and mTOR signaling pathways.^15^ Consistently, in our present study, we verified that the cytoplasmic expression of MARCH8 in NSCLC tissues was lower than that in normal lung tissues. We also confirmed that MARCH8 expression was distinctly correlated with tumor differentiation, histological type, and LNM status in NSCLC patients. A lower MARCH8 expression was correlated with poorer OS in NSCLC patients, suggesting that MARCH8 could potentially serve as an independent prognostic biomarker for NSCLC.

Previous studies have proved that tumor-infiltrating lymphocyte is an independent predictor of cancer patient prognosis and immunotherapeutic efficacy.^30,31^ MARCH8 has been reported to mediate the degradation of most transmembrane proteins such as immunomodulatory receptors MHC-II,^32^ suggesting the potential role of MARCH8 in immune regulation.^33^ And MARCH8 could ubiquitinate and degrade a nonmembrane protein, STAT3, for proteosome-dependent degradation and suppression of tumor metastasis in breast cancer cells.^13^ Our study demonstrated that MARCH8 expression related with tumor cell immune infiltration across cancer types. MARCH8 expression was significantly related to CD4+ T memory resting cells, B naive cells, and macrophages in most cancers, which proved the potential immune function of MARCH8 across cancer types. These results highlight the promising potential of MARCH8 in cancer immunotherapy research.

However, despite our systematic and comprehensive pan-cancer analysis on MARCH8, our study has some limitations. On the one hand, the data from different databases exist differences and lack specificity, which might cause background heterogeneity. We thus need further studies of larger sample sizes to further confirm the findings. On the other hand, we also need more in vivo/in vitro experiments to increase the credibility of our results on the potential function of MARCH8.

## Conclusions

In summary, our study first described the expression patterns, clinical relevance, and prognostic value of MARCH8 in cancers, especially the function in the progression of NSCLC disease. We figured out the potential of MARCH8 as biomarkers in tumor development, diagnosis, prognosis, and TME in cancers. Low expression of MARCH8 could act as an independent prognostic marker for NSCLC patients. Together, targeting MARCH8 might provide a promising potential in tumor development and treatment research.

## Abbreviations

ACC: Adrenocortical carcinoma
ALL: Acute Lymphoblastic Leukemia
BLCA: Bladder Urothelial Carcinoma
BRCA: Breast invasive carcinoma
CESC: Cervical squamous cell carcinoma and endocervical adenocarcinoma
CHOL: Cholangiocarcinoma
COAD: Colon adenocarcinoma
COADREAD: Colon adenocarcinoma/Rectum adenocarcinoma Esophageal carcinoma
DLBC: Lymphoid Neoplasm Diffuse Large B-cell Lymphoma
ESCA: Esophageal carcinoma
FPPP: FFPE Pilot Phase II
GBM: Glioblastoma multiforme
GBMLGG: Glioma
HNSC: Head and Neck squamous cell carcinoma
KICH: Kidney Chromophobe
KIPAN: Pan-kidney cohort (KICH+KIRC+KIRP)
KIRC: Kidney renal clear cell carcinoma
KIRP: Kidney renal papillary cell carcinoma
LAML: Acute Myeloid Leukemia
LGG: Brain Lower Grade Glioma
LIHC: Liver hepatocellular carcinoma
LUAD: Lung adenocarcinoma
LUSC: Lung squamous cell carcinoma
MESO: Mesothelioma
NB: Neuroblastoma
OS: Osteosarcoma
OV: Ovarian serous cystadenocarcinoma
PAAD: Pancreatic adenocarcinoma
PCPG: Pheochromocytoma and Paraganglioma
PRAD: Prostate adenocarcinoma
READ: Rectum adenocarcinoma
SARC: Sarcoma
STAD: Stomach adenocarcinoma
SKCM: Skin Cutaneous Melanoma
STES: Stomach and Esophageal carcinoma
TGCT: Testicular Germ Cell Tumors
THCA: Thyroid carcinoma
THYM: Thymoma
UCEC: Uterine Corpus Endometrial Carcinoma
UCS: Uterine Carcinosarcoma
UVM: Uveal Melanoma
WT: High-Risk Wilms Tumor

## Supplementary Material

Supplemental Material

Table S1 Re.docx

Figure S1.png

Figure S2.png

## Data Availability

The original contributions presented in the study are included in the article.
