# Supplementary material for: A pan-cancer analysis of MARCH8: molecular characteristics, clinical relevance, and immuno-oncology features
Source: Cancer Biol Ther. 2025 Jan 29;26(1):2458773. doi: 10.1080/15384047.2025.2458773 (PMC11784653; doi:10.1080/15384047.2025.2458773)
Supplement: Supplemental Material [file KCBT_A_2458773_SM3452.docx]

**Supplementary Material**

Figure S1. The relationship between MARCH8 expression and different clinical stages (A) and grades (B) across cancer types.

Figure S2. The relationship between MARCH8 expression and DFI (A) and DSS (B) across cancer types, using univariate survival analysis.

Table S1. Cancer names and abbreviations included in the TCGA database.
