# Supplementary material for: A pan-cancer analysis of MARCH8: molecular characteristics, clinical relevance, and immuno-oncology features
Source: Cancer Biol Ther. 2025 Jan 29;26(1):2458773. doi: 10.1080/15384047.2025.2458773 (PMC11784653; doi:10.1080/15384047.2025.2458773)
Supplement: Table S1 Re.docx [file KCBT_A_2458773_SM5483.docx]

| Abbreviations | Cancer names | Abbreviations | Cancer names |
| --- | --- | --- | --- |
| ACC | Adrenocortical carcinoma | LUAD | Lung adenocarcinoma |
| ALL | Acute Lymphoblastic Leukemia | LUSC | Lung squamous cell carcinoma |
| BLCA | Bladder Urothelial Carcinoma | MESO | Mesothelioma |
| BRCA | Breast invasive carcinoma | NB | Neuroblastoma |
| CESC | Cervical squamous cell carcinoma and endocervical adenocarcinoma | OS | Osteosarcoma |
| CHOL | Cholangiocarcinoma | OV | Ovarian serous cystadenocarcinoma |
| COAD | Colon adenocarcinoma | PAAD | Pancreatic adenocarcinoma |
| COADREAD | Colon adenocarcinoma/Rectum adenocarcinoma Esophageal carcinoma | PCPG | Pheochromocytoma and Paraganglioma |
| DLBC | Lymphoid Neoplasm Diffuse Large B-cell Lymphoma | PRAD | Prostate adenocarcinoma |
| ESCA | Esophageal carcinoma | READ | Rectum adenocarcinoma |
| FPPP | FFPE Pilot Phase II | SARC | Sarcoma |
| GBM | Glioblastoma multiforme | STAD | Stomach adenocarcinoma |
| GBMLGG | Glioma | SKCM | Skin Cutaneous Melanoma |
| HNSC | Head and Neck squamous cell carcinoma | STES | Stomach and Esophageal carcinoma |
| KICH | Kidney Chromophobe | TGCT | Testicular Germ Cell Tumors |
| KIPAN | Pan-kidney cohort (KICH+KIRC+KIRP) | THCA | Thyroid carcinoma |
| KIRC | Kidney renal clear cell carcinoma | THYM | Thymoma |
| KIRP | Kidney renal papillary cell carcinoma | UCEC | Uterine Corpus Endometrial Carcinoma |
| LAML | Acute Myeloid Leukemia | UCS | Uterine Carcinosarcoma |
| LGG | Brain Lower Grade Glioma | UVM | Uveal Melanoma |
| LIHC | Liver hepatocellular carcinoma | WT | High-Risk Wilms Tumor |

Table S1. Cancer names and abbreviations included in the TCGA database.
