# Supplementary figures and images for: A pan-cancer analysis of MARCH8: molecular characteristics, clinical relevance, and immuno-oncology features
Source: Cancer Biol Ther. 2025 Jan 29;26(1):2458773. doi: 10.1080/15384047.2025.2458773 (PMC11784653; doi:10.1080/15384047.2025.2458773)

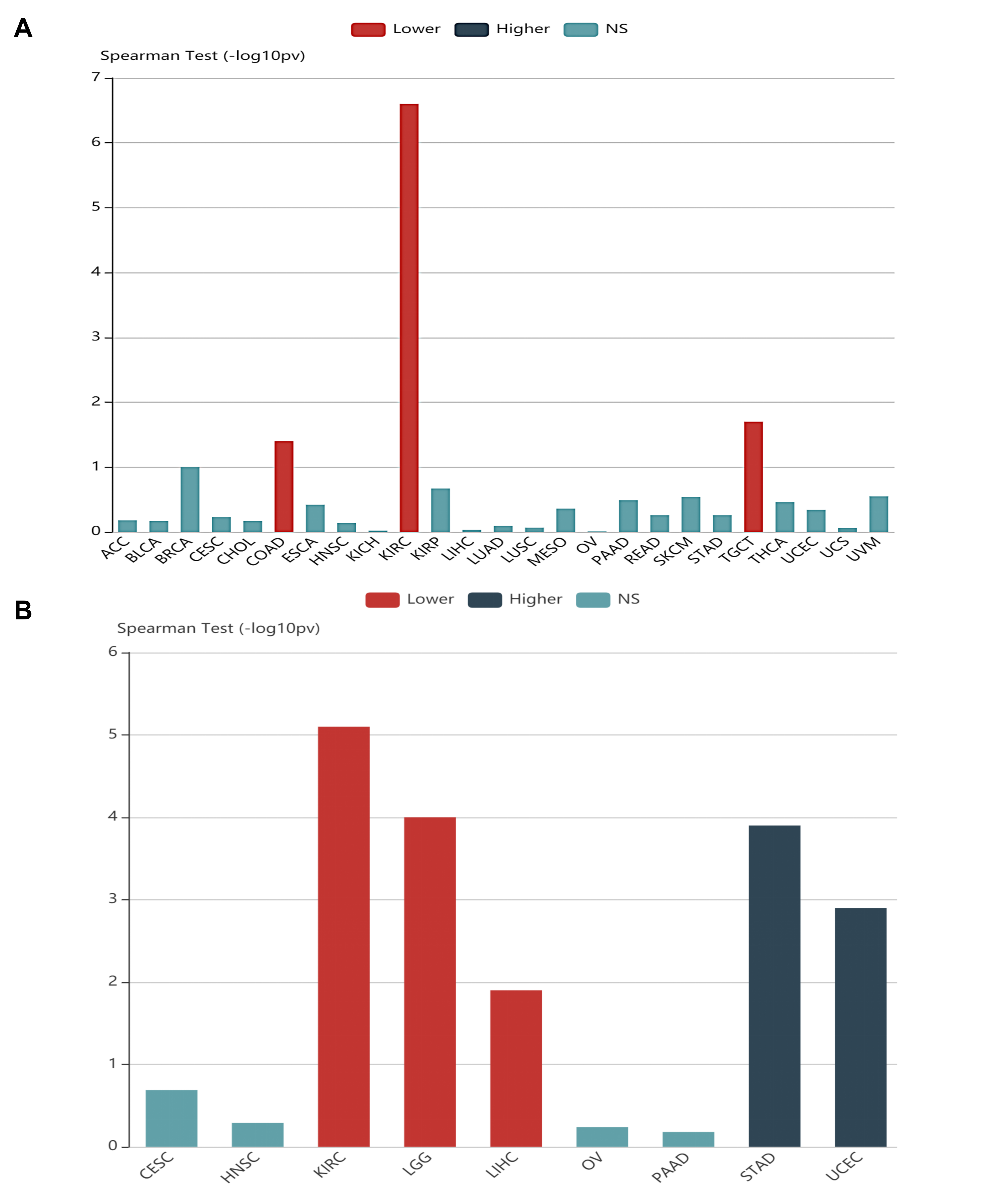

Supplement: Figure S1.png [file KCBT_A_2458773_SM5482.png]

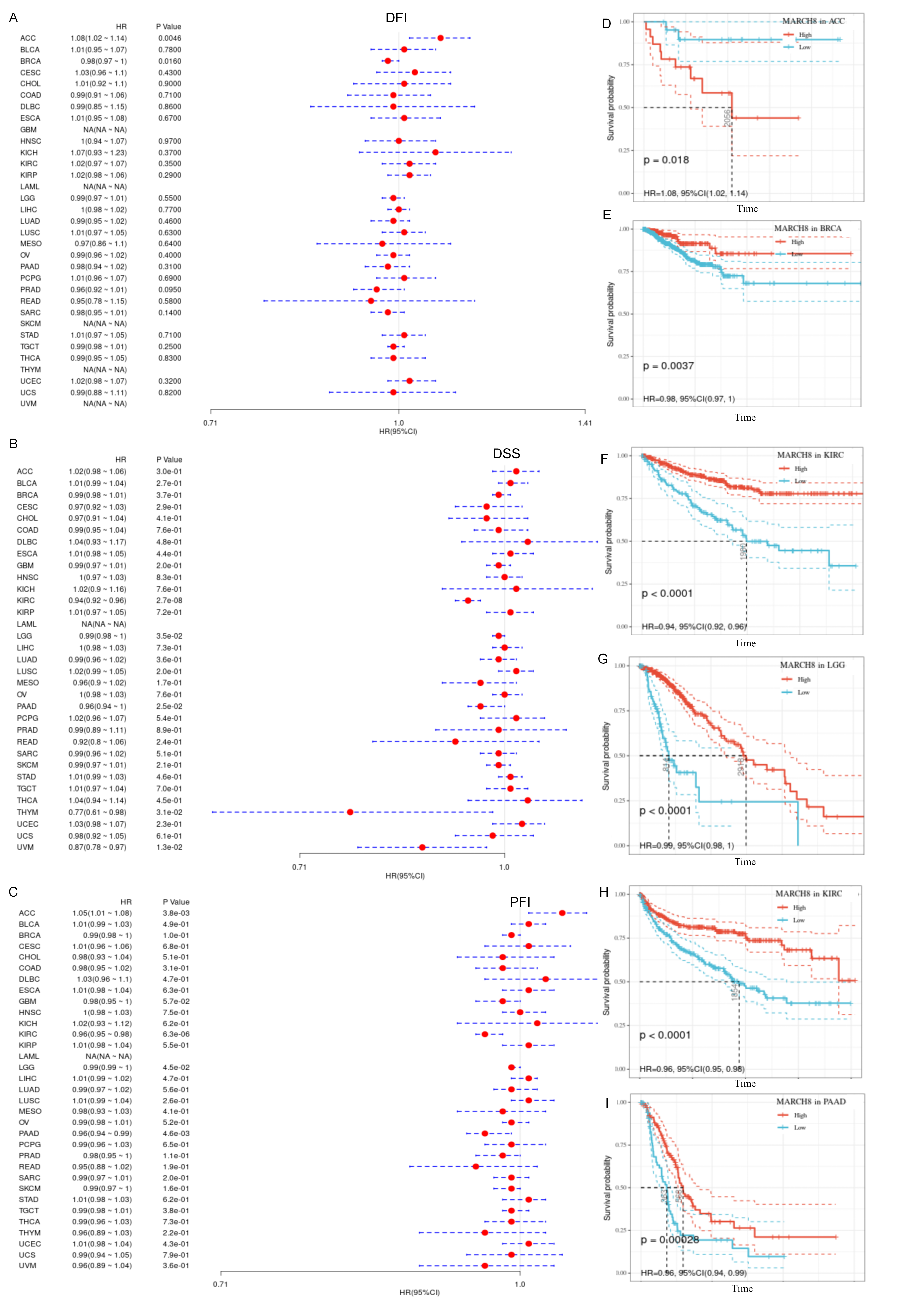

Supplement: Figure S2.png [file KCBT_A_2458773_SM5481.png]
